# Evolution of *Bordetella pertussis* over a 23-year period in France, 1996 to 2018

**DOI:** 10.2807/1560-7917.ES.2021.26.37.2001213

**Published:** 2021-09-16

**Authors:** Valérie Bouchez, Sophie Guillot, Annie Landier, Nathalie Armatys, Soraya Matczak, the French pertussis microbiology study group, Julie Toubiana, Sylvain Brisse, French pertussis microbiology study group, French pertussis microbiology study group, Nathalie Brieu, Farida Hamdad, Marie Kempf, Hélène Pailhoriès, Cécile Jensen, Philippe Lehours, Jennifer Guiraud, Hervé Le Bars, Christophe Isnard, Nathalie Wilhelm, Alain Le Coustumier, Julien Delmas, Dominique De Briel, Laurent Souply, Saïd Aberrane,, Marie Coudé, Fabien Garnier, Ghislaine Descours, Hélène Jean-Pierre, Corentine Alauzet, Sophie-Anne Gibaud, Stéphane Bonacorsi, Lucien Brasme, Ludovic Lemée, Christelle Koebel, Philippe Lanotte, Stéphane Bland, Hélène Petitprez, Didier Raffenot, Marion Levast, Florence Doucet-Populaire, Nadège Bourgeois-Nicolaos, Christophe Burucoa, Florence Grattard, Stéphanie Marque-Juillet

**Affiliations:** 1 Institut Pasteur, Biodiversity and Epidemiology of Bacterial Pathogens, Paris, France; 2 Institut Pasteur, National Reference Center for Whooping Cough and other Bordetella infections, Paris, France; 3 Sorbonne Université, Collège doctoral, Paris, France; 4 The members of the group are listed under Investigators; 5 Université de Paris, Department of General Pediatrics and Pediatric Infectious Diseases, Hôpital Necker–Enfants Malades, APHP, Paris, France

**Keywords:** *Bordetella pertussis*, vaccine antigen deficiency, pertactin, fimbriae serotype, population evolution

## Abstract

**Background:**

*Bordetella pertussis* is the main agent of whooping cough. Vaccination with acellular pertussis vaccines has been largely implemented in high-income countries. These vaccines contain 1 to 5 antigens: pertussis toxin (PT), filamentous haemagglutinin (FHA), pertactin (PRN) and/or fimbrial proteins (FIM2 and FIM3). Monitoring the emergence of *B. pertussis* isolates that might partially escape vaccine-induced immunity is an essential component of public health strategies to control whooping cough.

**Aim:**

We aimed to investigate temporal trends of fimbriae serotypes and vaccine antigen-expression in *B. pertussis* over a 23-year period in France (1996–2018).

**Methods:**

Isolates (n = 2,280) were collected through hospital surveillance, capturing one third of hospitalised paediatric pertussis cases. We assayed PT, FHA and PRN production by Western blot (n = 1,428) and fimbriae production by serotyping (n = 1,058). Molecular events underlying antigen deficiency were investigated by genomic sequencing.

**Results:**

The proportion of PRN-deficient *B. pertussis* isolates has increased steadily from 0% (0/38) in 2003 to 48.4% (31/64) in 2018 (chi-squared test for trend, p < 0.0001), whereas only 5 PT-, 5 FHA- and 9 FIM-deficient isolates were found. Impairment of PRN production was predominantly due to IS*481* insertion within the *prn* gene or a 22 kb genomic inversion involving the *prn* promoter sequence, indicative of convergent evolution. FIM2-expressing isolates have emerged since 2011 at the expense of FIM3.

**Conclusions:**

*B. pertussis* is evolving through the rapid increase of PRN-deficient isolates and a recent shift from FIM3 to FIM2 expression. Excluding PRN, the loss of vaccine antigen expression by circulating *B. pertussis* isolates is epidemiologically insignificant.

## Introduction

*Bordetella pertussis* is the main causative agent of whooping cough, a severe respiratory infection in humans [1]. Whole-cell pertussis vaccines (wPV) have been used on a large scale since 1959 and elicited a very strong decrease in disease incidence [2]. Despite their effectiveness, the use of wPV raised concerns about their side effects and production quality and they were replaced in many high-income countries by acellular pertussis vaccines (aPV). The aPV formulations contain one to five antigenic proteins of *B. pertussis* corresponding to virulence factors, including pertussis toxin (PT) and optionally filamentous haemagglutinin (FHA), pertactin (PRN) and fimbrial proteins (FIM2 and FIM3). In France, aPV were first introduced in 1998 as a booster for 11–13-year-old children, and progressively replaced wPV for prime vaccination from 2000. wPV were discontinued in 2004 for prime vaccination and in 2006 for all vaccinations (prime and boosters). Most aPV formulations now available in France contain two, three or five vaccine antigens (Supplementary Table S1).

Previous research has provided strong evidence that the evolution of *B. pertussis* populations is being driven by the selective pressure imposed by vaccine-induced immunity. Mutations in genes encoding aPV antigens (mainly *ptx*A, the structural gene for PT subunit 1, and *fim3*) and their promoters (*ptx*P) arose already in the period during which wPV were used, and allelic variants (*ptx*A1 and *ptx*P3) that differ from those of vaccine strains increased in frequency [3].

Besides antigen sequence divergence, escape from vaccine-induced immunity may result from the loss of production of antigens altogether. This phenomenon has been most observed for the antigen PRN, an outer membrane protein that promotes adhesion of *B. pertussis* to host epithelial cells [4]. PRN-deficient *B. pertussis* isolates have progressively increased in frequency in countries where aPV are used including France, Italy, Japan and the United States (US) [5], whereas no or very few PRN-negative isolates have been reported in countries that have continued the use of wPV, such as Iran [6]. Moreover, the proportion of PRN-negative *B. pertussis* isolates observed in a given country correlates positively with the time elapsed since the transition from wPV to aPV [7,8]. Finally, a reversion to aPV that do not contain PRN was associated with reversal of the evolutionary trend towards PRN-negative *B. pertussis* populations, as observed in Japan [9].

*B. pertussis* isolates lacking antigens other than PRN have been reported including FHA and PT, the major toxin of *B. pertussis,* although less frequently [10-12]. Although they are only contained in some aPV formulations, fimbrial proteins FIM2 and FIM3 are also important *B. pertussis* antigens [13]. Expression of fimbriae is governed by the regulatory *bvgAS* two component system and by the length of a poly(C) homopolymeric tract located in the promoter of genes *fim2* and *fim3* [13]. Usually either FIM2 or FIM3 is produced [13], sometimes both, but *B. pertussis* isolates that produce neither FIM2 nor FIM3 have been reported in Norway [7], Japan [14] and Canada [15].

During the pre-vaccine era, FIM2-expressing strains were predominant primarily in unvaccinated populations, as observed in the United Kingdom (UK) in 1920–56 when FIM2-expressing strains represented 58% of isolates [13]. Over time, FIM3-expressing strains have become highly predominant, as observed in Europe since 1998 or in Japan in the last two decades [7,13,14,16]. Shifts in the predominant fimbriae serotypes have been observed in different countries but a causal link with vaccination using FIM antigens is uncertain [13].

The discovery of *B. pertussis* isolates that do not express vaccine antigens has triggered concerns about the future effectiveness of aPV. A shift in FIM serotypes might also affect vaccine effectiveness, even though these two antigens are always simultaneously present or absent in vaccine formulations [13]. An evolutionary trend towards the loss of virulence factor expression is also of high clinical significance and may affect the pathogenesis of pertussis disease. The aim of this work was to define vaccine antigen production and fimbriae serotypes in a large collection of *B. pertussis* clinical isolates from France and to analyse temporal trends over a 23-year period of continuous surveillance.

## Methods

### *Bordetella pertussis* isolate collection

Since 1996, the French surveillance system of whooping cough is built on a sentinel hospital-based voluntary surveillance network (RENACOQ), coordinated by Public Health France, and is estimated to capture approximately one third of paediatric cases hospitalised with pertussis disease [17]. The National Reference Center (NRC) for Whooping Cough and other *Bordetella* infections at Institut Pasteur in Paris collects, isolates, identifies and archives *B. pertussis* clinical isolates captured through RENACOQ. In addition, several other French hospitals contribute to surveillance by referring their clinical isolates to the NRC. Isolates are mainly collected from children, but occasionally from their close contacts (older siblings, parents or grandparents).

### *Bordetella pertussis* microbiological characterisation

Bacterial samples were grown at 36°C for 72 h on Bordet Gengou Agar (BGA, Becton Dickinson, Le Pont de Claix, France) supplemented with 15% defibrinated sheep blood (ThermoFisher Diagnostics, Dardilly, France) and stored in BSA/saccharose–phosphate–glutamate solution at −80°C. All isolates (n = 2,280) were characterised using classical bacteriological methods, including observation of haemolysis, oxidase and urease tests. Identification was performed using the API 20 system (BioMerieux, Marcy-l‘Etoile, France) until 2015, when it was replaced by matrix assisted laser desorption ionization-time of flight (MALDI-TOF) mass spectrometry (Bruker, Billerica, Massachusetts, United States).

### Antigen characterisation

Production of PT, FHA and PRN in culture was assessed by Western blot on a random selection of isolates collected between 1996 and 2006 and on all isolates collected since 2007 (1,428/2,280). Serotyping to detect fimbrial proteins FIM2 and FIM3 was performed on all isolates collected from 2006 (1,058/2,280). 

Isolates were sub-cultured for 24 h in BGA medium (for Western blot or DNA extraction) or in charcoal agar (for serotyping). Bacteria were suspended in physiological salt and adjusted to an optical density (OD) at 650 nm of 1 (OD1), which was further used for microbiological characterisation. Western blot analyses were performed as previously described [18]. Serotyping was performed by agglutination tests following standard recommendations [16].

### DNA preparation and sequencing

A fraction (400 μL) of each OD1 suspension was pelleted and used for genomic DNA preparation as previously described [19]. Whole genome sequencing was performed using the Illumina NextSeq 500 system (Illumina, San Diego, California, US) at the Mutualized Platform for Microbiology of Institut Pasteur and used for de novo assembly, as previously detailed [19]. Raw FASTQ data from vaccine antigen-deficient isolates are available in the European Nucleotide Archive (https://www.ebi.ac.uk/ena; Projects: PRJEB21744 and PRJEB42353).

### Mutations analysis and genotyping

Sequence analysis was performed on genes *prn*, *fhaB*, *ptx* and *tcfA* from de novo assembled genomes using Basic Local Alignment Search Tool (blastN; https://blast.ncbi.nlm.nih.gov) with either Tohama (NC_002929/BX470248) or B1917 (CP009751) gene sequences as queries. Insertion sequence (IS) elements were searched using IS_mapper/0.1.5.1 (https://github.com/jhawkey/IS_mapper).

### Case definitions and clinical characteristics

General and clinical characteristics (i.e. age in months, vaccine status, symptoms, need for hospitalisation and intensive care support, leukocytosis, death attributable to pertussis) of cases corresponding to each *B. pertussis* isolate were collected. Cases were defined according to the national [20] or EU case definition [21]. For cases who represent the most vulnerable population (< 6 months of age), we characterised the severity of pertussis disease. A severe case was defined as an infant requiring intensive care support or who died from pertussis. A mild case was defined as a symptomatic infant with a confirmed case of pertussis who was not hospitalised or was hospitalised without any need for intensive care support. 

### Statistical analyses

The frequencies of PRN-negative and FIM2 phenotypes among *B. pertussis* isolates were calculated and tested for temporal trends during the study period (between 1996 and 2018 for PRN status, and between 2006 and 2018 for FIM2 status), using the chi-squared test for trend.

We compared clinical severity among pertussis cases caused by FIM2 and FIM3 isolates in the infant population (< 6 months of age) using the chi-squared test. Statistical significance was taken as p value < 0.05. Data analyses were performed using IBM SPSS statistics, version 21.0 (IBM, New York, New York, US).

### Ethical statement

All French bacteriological samples and associated clinical data are collected, coded, shipped, managed and analysed according to the National Reference Centre protocols that received approval by French supervisory ethics authority (CNIL, n°1474593).

## Results

### Temporal distribution of *Bordetella pertussis* isolates

**Figure 1:**
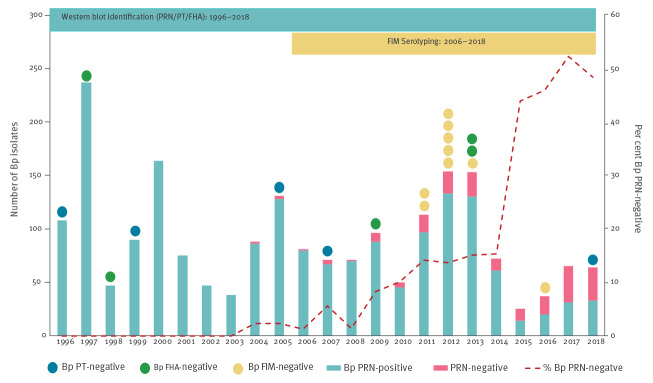
Number of *Bordetella pertussis* isolates and pertussis vaccine antigen-negative isolates collected per year, France, 1996–2018 (n = 2,280)

A total of 2,280 isolates were collected continuously between 1996 and 2018. As illustrated in Figure 1, the number of isolates collected per year fluctuated, with peaks observed in 1997, 2000, 2005, 2012–13 and 2017–18, i.e. every 3 to 5 years corresponding to the previously described cyclical pattern of pertussis disease [22]. The most recent increase of *B. pertussis* isolates, observed in 2017–18, showed a lower peak than the previous one.

Bp: *Bordetella pertussis*; FHA: filamentous haemagglutinin; FIM: fimbrial protein; PRN: pertactin; PT: pertussis toxin.

Coloured bars represent the total number of *B. pertussis* isolates per year. Turquoise sections: PRN-producing isolates. Pink sections: PRN-negative isolates. Dashed red line: per cent of PRN-negative isolates. Green circles: FHA-negative isolates. Blue circles: PT-negative isolates. Yellow circles: FIM-negative isolates. Please note that the dots represent individual strains that are already counted in the bars and are not to be interpreted with regards to the Y-axis scale.

The case age, available for 2,026/2,280 isolates, ranged from 1 day to 93 years (median age: 3.7 months). For each year examined, the median age of cases was between 2.9 and 5.1 months, except in 2016 when it was higher (9.9 months). The sex ratio (female/male) for cases was 1.16, based on 1,972 isolates for which the information was available.

### Vaccine antigen-deficient isolates

**Figure 2:**
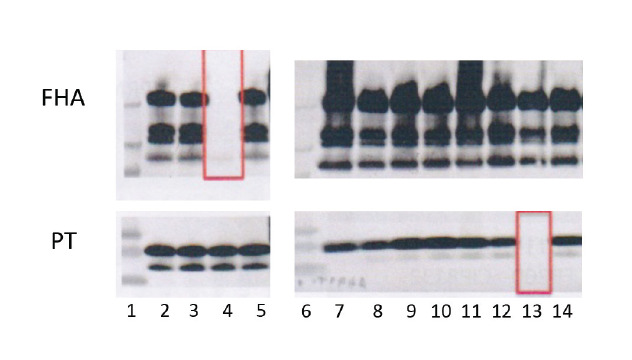
Identification of filamentous haemagglutinin- and pertussis toxin-negative *Bordetella pertussis* isolates by Western blot, France, 1996 to 2018 (n = 1,428)

FHA: filamentous haemagglutinin; PT: pertussis toxin.

We observed very few PT, FHA or FIM-negative isolates (Figure 2; Table 1). Five PT-negative isolates (0.35%; 5/1,428 of isolates identified using Western blot) were identified, of which two (FR3469 and FR3749) were previously reported [10,23]. The most recent PT-negative isolate, collected in 2018, was also PRN-negative. Five isolates that produce very little or no FHA (0.35%; 5/1,428 of isolates tested using Western blot) were observed, among which FR4624 was previously described [23,24]. Two FHA-negative isolates were also negative for PRN (FR4624 and FR5683). For another isolate (FR5771), FHA production was very weak rather than fully negative. Finally, of 1,058 isolates tested using serotyping, nine (0.63%) produced neither FIM2 nor FIM3 and are reported for the first time here (Table 1). These FIM-deficient isolates produced PRN, FHA and PT.

Representative Western blot screening for FHA (upper panel: expected size 240 kDa) and PT (lower panel: expected size 28 kDa). Lanes were loaded with 10 μl of either Laemmli-treated samples or a molecular weight ladder. Lanes 1 and 6: 10–250 kDa ladder. Lane 4: FHA-deficient isolate profile (FR0432). Lane 13: PT-deficient isolate profile (FR0270). Other lanes: randomly selected samples from co-circulating *Bordetella pertussis* isolates.

**Table 1:** Characteristics of *Bordetella pertussis* isolates deficient for the production of filamentous haemagglutinin, pertussis toxin or fimbriae and corresponding clinical data of pertussis cases, France, 1996–2018 (n = 19)

Isolate characteristics			Clinical characteristics of pertussis cases					
Name	Deficient antigen(s)	Year	Case age (months)	Vaccine status	Symptoms^a^	Hospitalisation	Leukocytosis	Ref.
FR0270	PT-	1996	NA	NA	NA	Yes	NA	*This study*
FR0694	PT-	1999	2.6	Not vaccinated	Paroxysmal cough	Yes	NA	*This study*
FR3469	PT-	2005	2.0	Not vaccinated	Paroxysmal cough for 15 days Bradycardia/cyanosis	Yes	NA	[23]
FR3749	PT-	2007	3.2	aPV, 1 dose	Paroxysmal cough	Yes	No	[10]
FR6595	PT-/PRN-	2018	39	aPV, 3 doses	Paroxysmal cough for 15 days	No	NA	NA
FR0432	FHA-	1997	NA	NA	NA	NA	NA	*This study*
FR0658	FHA-	1998	4.6	NA	NA	NA	NA	*This study*
FR4624	FHA-/PRN-	2009	2.1	Not vaccinated	Paroxysmal cough for 21 days	No	No	[23]
FR5771	FHA+/ −	2013	2.5	Not vaccinated	Respiratory distress, encephalopathy	Yes (paediatric intensive care)	Leukocytosis^b^	*This study*
FR5683	FHA-/PRN-	2013	2.6	aPV, 1 dose	Paroxysmal cough, cyanosis	Yes	No	*This study*
FR4922	FIM2-/FIM3-	2011	1.9	Not vaccinated	Paroxysmal cough	Yes	NA	*This study*
FR4925	FIM2-/FIM3-	2011	0.9	Not vaccinated	Paroxysmal cough	Yes	NA	*This study*
FR5134	FIM2-/FIM3-	2012^c^	1.4	Not vaccinated	Paroxysmal cough	Yes	NA	*This study*
FR5135	FIM2-/FIM3-	2012	1.3	Not vaccinated	Paroxysmal cough, hypotonia	Yes	NA	*This study*
FR5136	FIM2-/FIM3-	2012	1.7	Not vaccinated	Paroxysmal cough	Yes	NA	*This study*
FR5139	FIM2-/FIM3-	2012	1.0	Not vaccinated	Paroxysmal cough	Yes	NA	*This study*
FR5167	FIM2-/FIM3-	2012	0.9	Not vaccinated	Paroxysmal cough, hypotonia	Yes	NA	*This study*
FR5763	FIM2-/FIM3-	2013	3.6	Not vaccinated	Paroxysmal cough, cyanosis	Yes	NA	*This study*
FR6063	FIM2-/FIM3-	2016	70	aPV, 4 doses	Paroxysmal cough for 15 days	No	NA	*This study*

aPV: acellular pertussis vaccine; FHA: filamentous haemagglutinin; FIM: fimbrial protein; NA: non-available data; PRN: pertactin; PT: pertussis toxin; Ref.: references.

^a^ For cases with paroxysmal cough where number of days was not defined, the duration was unknown.

^b^ Leukocytosis: white blood cell count was 57,000/mm^3^ (norm: 5,500–15,500/mm^3^).

^c^ FIM2-/FIM3- isolates from 2012 were not related as they were collected in children from different cities.

Clinical data for pertussis cases corresponding to the identified vaccine antigen-deficient isolates were reviewed (Table 1). Infections caused by PT-negative isolates (n = 5) occurred in children aged 2.0 to 39 months. Of the cases with PT-negative isolates with available clinical data, two children had not begun the recommended vaccine course and four were hospitalised with mild illness. The FHA-weak isolate FR5771 was collected from an unvaccinated 2.5-month old infant with a fulminant disease with respiratory distress syndrome, encephalopathy and leukocytosis requiring leukodepletion. FIM-negative isolates were from cases with mild pertussis illness who were very young and mostly unvaccinated (median age: 1.6 months (range: 0.9–70)) (Table 1).

PRN was by far the vaccine antigen most frequently deficient, as there were 188 PRN-negative isolates (13.2%) of 1,428 tested (Supplementary Table S3). During the 10-year pre-aPV period (1996–2006), only six PRN-negative isolates were observed, all of which were collected toward the end of the period (i.e. between 2004 and 2006). On the contrary, 182 PRN-negative isolates were found during the 11-year post-aPV period (2007–18). The proportion increased steadily from 5.6% in 2007 to 48.4% in 2018 (chi-squared test for trend, p < 0.0001) (Figure 1).

### Analysis of genetic events leading to impairment of pertactin, pertussis toxin and filamentous haemagglutinin production

**Figure 3:**
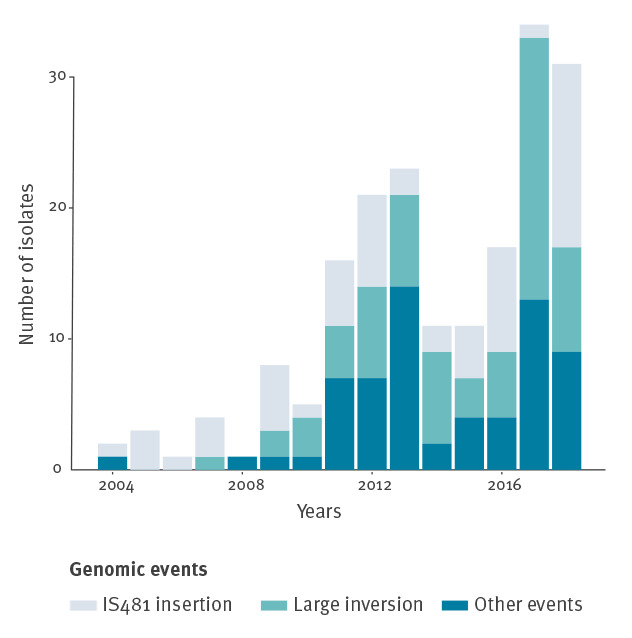
Genomic events leading to loss of pertactin expression in *Bordetella pertussis* isolates per year, France, 2004–2018 (n = 188)

All vaccine antigen-negative *B. pertussis* isolates (n = 198) were investigated for production-disrupting genetic events at the corresponding gene loci (Supplementary Table S2). For isolates with PRN production disruption, 30 distinct events were observed, corresponding to various insertions, deletions, inversions or mutations. Two events were observed much more frequently than the others: an IS*481* insertion at *prn* position 1613–14 representing 31.2% (59/188) of PRN-negative isolates, and a large 22 kb inversion involving the *prn* promoter region (35.5%; 67/188). Their temporal evolution (Figure 3) showed that both events contributed importantly to the steady increase of PRN-deficient *B. pertussis*.

Gray bars: number of PRN-negative isolates with an IS481 insertion within the *prn* gene. Turquoise bars: number of PRN-negative isolates with a 22 kb inversion. Dark blue bars: number of isolates with other genomic events impairing PRN production.

Among the PT-negative isolates (n=5), three different events were observed. A deletion of the entire PT operon was observed for three isolates (FR0270, FR0694, FR3749), similar to previous reports [10,11], whereas a seven nucleotide deletion in *ptx*S4 and a one nucleotide deletion within *ptx*S1 subunits, respectively, explained the PT-negative phenotype in isolates FR3469 and FR6595.

Of the FHA-negative isolates (n=5), two main insertion events were uncovered. First, a 1 bp deletion in position 1087 of the *fhaB* gene for isolates FR0658 and FR5771 was identified, as previously reported [12,23]. Second, the insertion of an IS*481* element took place within the *fhaB* gene in isolates FR0432, FR4624 and FR5683.

### Temporal trends in the production of fimbrial proteins and association with clinical severity

**Figure 4:**
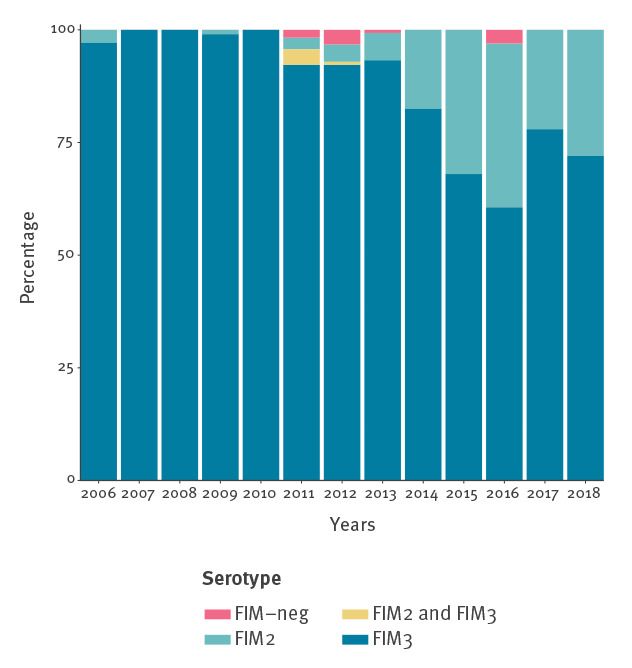
Evolution of fimbrial protein production by *Bordetella pertussis* isolates per year, France, 2006–2018 (n = 1,058)

FIM: fimbrial protein; neg: negative.

All isolates collected since 2006 (n = 1,058) were serotyped to detect the production of fimbrial proteins FIM2 and FIM3. A large majority of isolates (90.1%; 953/1,058) produced FIM3. However, starting in 2011, a continuous increase of the proportion of isolates producing FIM2 was observed (chi-squared test for trend p value: < 0.0001; Figure 4). FIM2 isolates represented 27.9% (19/68) of isolates collected in 2018 vs only 2.6% (3/116) in 2011. In addition, five isolates were identified that produced both FIM2 and FIM3. These atypical isolates, collected in 2011 and 2012, also produced PRN, FHA and PT. No significant difference in severity of pertussis infections was observed between infants < 6 months harbouring FIM2 (n = 63) and FIM3 producing (n = 103) isolates (42% (26/63) vs 36% (37/103) for severe disease; p value = 0.28).

Dark blue: per cent FIM3-producing isolates. Turquoise: per cent FIM2-producing isolates. Yellow: per cent FIM2- and FIM3-producing isolates. Pink: per cent FIM-negative isolates.

## Discussion

There is strong evidence that vaccine-induced immunity exerts a selective pressure on *B. pertussis*, leading to antigenic drift and the loss of expression of vaccine antigens [3,5,8]. The surveillance of antigen production by *B. pertussis* isolates aims at detecting the potential emergence and dissemination of *B. pertussis* strains evolving towards vaccine escape. Thus, it is critical to monitor the potential loss of effectiveness of pertussis vaccines in current use. Here, we leveraged the continuous French *B. pertussis* surveillance system, which has been in place and unchanged since 1996, to perform a comprehensive analysis of antigen expression in *B. pertussis* populations and its evolutionary trends over more than two decades.

Previous reports have described a few *B. pertussis* isolates that did not produce PT, FHA or FIM, but the epidemiological significance of these observations and their temporal trends remain elusive. PT is included in all aPV, either alone or together with one to four other antigens. Only four PT-negative isolates have been previously reported worldwide [10-12]. Here, we report three additional PT-negative isolates, from both unvaccinated and vaccinated symptomatic children. The loss of PT production might alter *B. pertussis* virulence, as suggested by cellular and mouse models [10].

Several FHA-deficient isolates have been previously reported from France [24], Sweden [25], US [12] and Australia [26]. Reduced FHA production has also been reported [12]. Here, we identified four additional isolates deficient in FHA production and one isolate with a reduced FHA production. The isolate with weak FHA production (FR5771) was collected from a case displaying serious complications observed in fulminant forms of pertussis. Similar isolates have been collected from children in the US with paroxysmal cough and apnoea; one of them also presented cyanosis and was hospitalised [12]. These data suggest that *B. pertussis* with altered FHA production might be able to cause a severe form of the disease. In murine models, both FHA and FHA/PRN-negative isolates are cytotoxic for macrophages, being lethal at high concentration [23], and were associated with increased expression of other vaccine antigens [24].

Double FIM-negative isolates have previously been reported in Japan [14], Canada [15] and Norway [7]. Here, we identified nine double-FIM-negative isolates. As these isolates produced PT, PRN and FHA, which are other *bvg*-regulated virulence factors, FIM2 and FIM3 production deficiency cannot be attributed to this regulator. Although fimbrial gene expression is affected by the length of C-stretch in the promotor [13], neither Sanger nor Illumina sequencing allowed the determination of the precise C-stretch length within *pfim2* and *pfim3*; this would require further investigations using a PCR/ligase detection reaction approach, for example. In France, vaccine-induced selective pressure directed at the fimbrial proteins may not be as strong as for other vaccine antigens, since only a few of the available vaccine formulations contain fimbriae, and one is mainly as a booster for children 11 to 13 years of age and adults (Supplementary Table S1). Most FIM-negative isolates were collected from unvaccinated cases with mild disease, raising the hypothesis that these isolates might be less virulent.

The observation that PT-, FHA- and FIM-negative isolates correspond to symptomatic cases with cough suggests their ability to induce pertussis-like symptoms. However, an alternative possibility is that antigen deficiency evolved in vitro, after isolation from the patient. This scenario would be consistent with the lack of reported transmission of PT-, FHA- and FIM-negative isolates. Multiple isolates from single cases, longitudinal sampling, carriage screening of contacts and metagenomics will be useful to investigate this hypothesis.

In contrast to the other four aPV antigens, nearly half of French *B. pertussis* isolates do not produce PRN. Several genetic events leading to PRN expression loss have been reported from different studies [5,27,28]. Here, we observed two main genomic events (IS*481* insertion or a large inversion) that led to impairment of PRN production, in agreement with observations in other world regions [7,27,29]. This pattern reflects convergent evolution and is further evidence that loss of expression of PRN is under strong selective pressure [30]. PRN loss was shown to confer a better fitness to *B. pertussis* in PRN-vaccinated mouse models [31,32] and to be more frequently observed among *B. pertussis* isolates from vaccinated cases [33]. This work demonstrates the continuous increase of the proportion of PRN-negative *B. pertussis* since 2004, a year that coincides closely with the change from wPV to aPV for prime vaccination in France. In regions of the US where aPV were introduced earlier (1991) [34], the proportion of PRN-negative isolates have reached > 80% [28]. In Europe, the highest proportions of PRN-negative isolates were found in Sweden (69%) and Italy (55%), where aPV were introduced in 1995–96, earlier than in other countries [7]. The observed continuous upward trend in the frequency of PRN-negative isolates in France and the observed effect of the duration of aPV usage in other countries are consistent with the long-term maintenance of a selective advantage of PRN-negative isolates. The frequency of PRN-negative isolates is therefore expected to further increase in the future, which may result in a lower efficacy of PRN-containing aPV against transmission.

Fimbriae serotypes of French isolates were previously reported for the 1992–2004 period, which showed that FIM3 predominated [16]. Here, we also found that FIM3 isolates were largely predominant. However, we observed a continuous and rapid increase in the frequency of FIM2 isolates since 2011. Shifts in predominant fimbriae serotypes have already been reported in the UK and in Finland [13,35]. aPV either contain both FIM2 and FIM3 or no FIM. Therefore, vaccine-induced selective pressure against FIM2 or FIM3 is expected to be similar. In contrast, as antibodies raised against FIM2 and FIM3 are not cross-reactive, negative frequency-dependent selection driven by immunity to natural infection or carriage might explain the FIM2/FIM3 shifts. We did not observe any difference of clinical severity between infections caused by FIM2 or FIM3 isolates. Given the fluctuation of their relative frequencies, FIM2- and FIM3-expressing isolates should be further investigated for possible specificities in pathogenesis, carriage and transmission.

One limitation of our study is that our sampling was derived from hospitalised cases of pertussis, mostly in young infants, which might introduce a bias and differ from pertussis surveillance isolates in other countries such as Australia or the US, where surveillance covers all ages rather than only children [2]. However, *B. pertussis* isolates from hospitalised infants are believed to be representative of the general *B. pertussis* population, as these isolates mix well phylogenetically [19] and, as most infections in young infants, originate from older members of the household [17]. Another limitation is that some diagnostic laboratories only perform RT-PCR-based diagnosis and do not try to cultivate *B. pertussis* anymore. Given the difficulty of isolating *B. pertussis* bacteria from nasopharyngeal aspirates, only samples with a high bacterial load can lead to a positive culture. Finally, we did not have full access to clinical data for all the isolates of our collection, impairing a more global clinical-based analysis.

## Conclusions

Our results demonstrate the rarity of PT- FHA- and FIM-negative isolates, consistent with literature. Based on more than two decades of continuous *B. pertussis* antigen production surveillance and on existing literature, we suggest that the occurrence of PT-, FHA- and FIM-negative *B. pertussis* isolates will remain of negligible epidemiological significance. In contrast, the rapid rise of PRN-negative and FIM2-expressing isolates calls for future prospective studies to define the significance of contemporaneous *B. pertussis* evolution on disease severity and vaccine effectiveness.

## Supplementary Data

330000 Supplement
