# Supplementary material for: Evolution of Bordetella pertussis over a 23-year period in France, 1996 to 2018
Source: Euro Surveill. 2021 Sep 16;26(37):2001213. doi: 10.2807/1560-7917.ES.2021.26.37.2001213 (PMC8447829; doi:10.2807/1560-7917.ES.2021.26.37.2001213)
Supplement: Supplement [file 20-01213_BRISSE_Supplement.pdf]

This supplementary material is hosted by Eurosurveillance as supporting information alongside the article "**Evolution of *Bordetella pertussis* in France over a 23-year period (1996-2018**) on behalf of the authors, who remain responsible for the accuracy and appropriateness of the content. The same standards for ethics, copyright, attributions and permissions as for the article apply.
Supplements are not edited by Eurosurveillance and the journal is not responsible for the maintenance of any links or email addresses provided therein

Table S1. Vaccines used in France
Quantities are given per dose (0.5 ml)

	**Prime vaccination**	**Boosters**		
**aPV-2 components PT+FHA**	Hexyon®	Tetratavac®	Pentavac®	
**PT**	25 µg	25 µg	25 µg	
**FHA**	25 µg	25 µg	25 µg	
Marketing Authorisation year	2013	1998	1998	
**aPV-3 components PT+FHA+PRN**	InfanrixHexa®	BoostrixTetra	InfanrixTetra®	InfanrixQuinta®
**PT**	25 µg	8 µg	25 µg	25 µg
**FHA**	25 µg	8 µg	25 µg	25 µg
**PRN**	8 µg	2.5 µg	8 µg	8 µg
Marketing Authorisation year	2000	2005	1996	1997
**aPV-5 components PT+PRN+FHA+FIM2+FIM3**	Vaxelis®	Repevax®		
**PT**	20 µg	2.5 µg		
**FHA**	20 µg	5 µg		
**PRN**	3 µg	3 µg		
**FIM2-FIM3**	5 µg	5 µg		
Marketing Authorisation year	2016	2002		

Table S2. Genomic events leading to production deficiency of pertactin, filamentous haemagglutinin or pertussis toxin

Antigen	Event type	Event	Genomic location	Reference	Number of isolates
**PRN**	**inversions**	22 kb large inversion within promoter of prn2	position -20892 to -75	Zeddeman, Eurosurveillance, 2014	66
		22 kb large inversion within promoter of prn2 + SNP position 1834	position -20892 to -75 + SNP 1834	this study	1
	**insertions**	insertion of IS481 within prn2	position 245-246	Zeddeman, Eurosurveillance, 2014	2
		G insertion within prn2	position 1185	Barkoff, Adv.Exp.Med. Biol, 2019	0
		insertion of IS481 within prn3	position 1598-1599	Xu, EID, 2019	0
		insertion of IS481 within prn1	position 1598-1599	Zeddeman, Eurosurveillance, 2014	0
		insertion of IS481 within prn2	position 1613-1614	Zeddeman, Eurosurveillance, 2014	58
		insertion of IS1002 within prn2	position 1613-1614	Xu, EID, 2019	0
		insertion of IS481 within prn2	position 2735-3736	Zeddeman, Eurosurveillance, 2014	1
	**deletions**	witihin promoter	position -2090 to -478	Barkoff, Adv.Exp.Med. Biol, 2019	0
		witihin promoter (prn3)	position -403 to -73	this study	1
		witihin promoter	position -283 to -40	Weigand, Genome Announcement, 2017	0
		witihin promoter	position -293 to -73	this study	1
		within promoter and first part of prn gene	position -1513 to 145	Weigand, Genome Announcement, 2017	0
		within promoter and first part of prn gene	position -1846 to 553	Zeddeman, Eurosurveillance, 2014	1
		within promoter and first part of prn gene	position -419 to 1325	this study	4
		within promoter and first part of prn gene	position -292 to 1340	Weigand, Genome Announcement, 2017	10
		within promoter and first part of prn gene	position -403 to 1608	this study	1
		within prn gene (84 bp deletion)	position 26 to 109	Barkoff, Adv.Exp.Med. Biol, 2019	0
		within prn gene (89 bp deletion at 5' of prn2)	nd	Zeddeman, Eurosurveillance, 2014	0
		within prn gene (49 bp deletion)	position 32 to 80	Zeddeman, Eurosurveillance, 2014	0
		within prn1 signal sequence (110 bp deletion)	position 0 to 110	Zeddeman, Eurosurveillance, 2014	2

		T deletion within prn2	position 628	this study	2
		T deletion within prn2	position 631-632	Barkoff, Adv.Exp.Med. Biol, 2019	0
		x deletion within prn2	position 666-667	Weigand, Genome Announcement, 2017	0
		G deletion (position 1494) an SNP position 1834 within prn2	position 1494-1832-1834	this study	3
		entire prn gene deletion	position 1098091 to 1100823 (Tohama)	Zeddeman, Eurosurveillance, 2014	0
		25 bp deletion in first repeat region of prn2	position 1043-1067	Zeddeman, Eurosurveillance, 2014	1
		4 bp deletion within prn2	position 2020-2023	Xu, EID, 2019	0
		14 bp deletion within end of prn2	postion 2734-2748	this study	1
	**point mutations**	SNP -162 A>G within prn2	position -162	this study	3
		SNP 223 C>T leading to stop codon within prn2	position 223	Zeddeman, Eurosurveillance, 2014	10
		SNP 760 C>T leading to stop codon within prn2	position 760	Zeddeman, Eurosurveillance, 2014	0
		SNP -308 G>A and G deletion (position 1016) within prn2	position -308 and 1016	this study	1
		SNP 1273 C>T leading to stop codon within prn2	position 1273	Zeddeman, Eurosurveillance, 2014	1
		SNP 1494 leading to stop codon within prn2	position 1494	Zeddeman, Eurosurveillance, 2014	1
		SNP 1834 T>A within prn2 and SNP G>T 1852	position 1834-1852	this study	4
		SNP 1852 G>T within prn2	position 1852	this study	3
		SNP 2077 G>T leading to stop codon within prn2	position 2077	Barkoff, Adv.Exp.Med. Biol, 2019	0
	**others**	4bp modification CCCA vs TTGT within prn2	position 1831	this study	2
**FHA**	**insertions/ deletions**	deletion/insertion in G-homopolymeric stretch	position 1087	Weigand, Infection & Immutnity, 2018	3
		insertion of IS481	position 3124	Weigand, Infection & Immutnity, 2018	2
		insertion of IS481	position 3096 - 7517	this study	1
		insertion of IS481	position 9865	Weigand, Infection & Immutnity, 2018	0

**PT**	**deletions**	28 kb *ptx-ptl* deletion	position 3986788 to 4014829 (Tohama)	Bouchez, Plos One, 2008/ Williams, EID, 2016	3
		T deleltion within ptxS1	position 47	this study	1
		7 bp deletion within ptxS4	position 45-51	this study	1

Table S3 : Accession Numbers for PRN-negative, PT-negative and FHA-negative Bp isolates, France, 1996-2018.

Name of isolate	Deficiency	Sample Accession No.	Project Accession No.
FR0270	PT-	ERS5513122	PRJEB42353
FR0694	PT-	ERS5513123	PRJEB42353
FR3469	PT-	ERS5513124	PRJEB42353
FR3749	PT-	ERS5513125	PRJEB42353
FR6595	PT-/PRN-	ERS5513126	PRJEB42353
FR0432	FHA-	ERS5513127	PRJEB42353
FR0658	FHA-	ERS5513128	PRJEB42353
FR4624	FHA-/PRN-	ERS5513129	PRJEB42353
FR5771	FHA-	ERS5513130	PRJEB42353
FR5683	FHA-/PRN-	ERS5513131	PRJEB42353
FR3098	PRN-	ERS5513132	PRJEB42353
FR3112	PRN-	ERS5513133	PRJEB42353
FR3276	PRN-	ERS5513134	PRJEB42353
FR3421	PRN-	ERS5513135	PRJEB42353
FR3424	PRN-	ERS5513136	PRJEB42353
FR3512	PRN-	ERS5513137	PRJEB42353
FR3693	PRN-	ERS5513138	PRJEB42353
FR3705	PRN-	ERS5513139	PRJEB42353
FR3708	PRN-	ERS5513140	PRJEB42353
FR3793	PRN-	ERS5513141	PRJEB42353
FR3925	PRN-	ERS5513142	PRJEB42353
FR4028	PRN-	ERS5513143	PRJEB42353
FR4068	PRN-	ERS5513144	PRJEB42353
FR4072	PRN-	ERS5513145	PRJEB42353
FR4591	PRN-	ERS5513146	PRJEB42353
FR4596	PRN-	ERS5513147	PRJEB42353
FR4611	PRN-	ERS5513148	PRJEB42353
FR4612	PRN-	ERS5513149	PRJEB42353
FR4671	PRN-	ERS5513150	PRJEB42353
FR4683	PRN-	ERS5513151	PRJEB42353
FR4684	PRN-	ERS5513152	PRJEB42353
FR4688	PRN-	ERS5513153	PRJEB42353
FR4698	PRN-	ERS5513154	PRJEB42353
FR4868	PRN-	ERS5513155	PRJEB42353
FR4873	PRN-	ERS5513156	PRJEB42353
FR4882	PRN-	ERS5513157	PRJEB42353
FR4913	PRN-	ERS5513158	PRJEB42353
FR4916	PRN-	ERS5513159	PRJEB42353
FR4923	PRN-	ERS5513160	PRJEB42353
FR4937	PRN-	ERS5513161	PRJEB42353

FR4947	PRN-	ERS5513162	PRJEB42353
FR4950	PRN-	ERS5513163	PRJEB42353
FR4952	PRN-	ERS5513164	PRJEB42353
FR4953	PRN-	ERS5513165	PRJEB42353
FR4963	PRN-	ERS5513166	PRJEB42353
FR4964	PRN-	ERS5513167	PRJEB42353
FR5001	PRN-	ERS5513168	PRJEB42353
FR5018	PRN-	ERS5513169	PRJEB42353
FR5027	PRN-	ERS5513170	PRJEB42353
FR5043	PRN-	ERS5513171	PRJEB42353
FR5126	PRN-	ERS5513172	PRJEB42353
FR5186	PRN-	ERS5513173	PRJEB42353
FR5187	PRN-	ERS5513174	PRJEB42353
FR5189	PRN-	ERS5513175	PRJEB42353
FR5224	PRN-	ERS5513176	PRJEB42353
FR5256	PRN-	ERS5513177	PRJEB42353
FR5257	PRN-	ERS5513178	PRJEB42353
FR5259	PRN-	ERS5513179	PRJEB42353
FR5261	PRN-	ERS5513180	PRJEB42353
FR5290	PRN-	ERS5513181	PRJEB42353
FR5299	PRN-	ERS5513182	PRJEB42353
FR5302	PRN-	ERS5513183	PRJEB42353
FR5308	PRN-	ERS5513184	PRJEB42353
FR5317	PRN-	ERS5513185	PRJEB42353
FR5345	PRN-	ERS5513186	PRJEB42353
FR5347	PRN-	ERS5513187	PRJEB42353
FR5363	PRN-	ERS5513188	PRJEB42353
FR5388	PRN-	ERS5513189	PRJEB42353
FR5391	PRN-	ERS5513190	PRJEB42353
FR5394	PRN-	ERS5513191	PRJEB42353
FR5433	PRN-	ERS5513192	PRJEB42353
FR5461	PRN-	ERS5513193	PRJEB42353
FR5465	PRN-	ERS5513194	PRJEB42353
FR5477	PRN-	ERS5513195	PRJEB42353
FR5478	PRN-	ERS5513196	PRJEB42353
FR5481	PRN-	ERS5513197	PRJEB42353
FR5490	PRN-	ERS5513198	PRJEB42353
FR5495	PRN-	ERS5513199	PRJEB42353
FR5506	PRN-	ERS5513200	PRJEB42353
FR5516	PRN-	ERS5513201	PRJEB42353
FR5563	PRN-	ERS5513202	PRJEB42353
FR5570	PRN-	ERS5513203	PRJEB42353
FR5579	PRN-	ERS5513204	PRJEB42353

FR5685	PRN-	ERS5513205	PRJEB42353
FR5686	PRN-	ERS5513206	PRJEB42353
FR5697	PRN-	ERS5513207	PRJEB42353
FR5713	PRN-	ERS5513208	PRJEB42353
FR5714	PRN-	ERS5513209	PRJEB42353
FR5742	PRN-	ERS5513210	PRJEB42353
FR5746	PRN-	ERS5513211	PRJEB42353
FR5772	PRN-	ERS5513212	PRJEB42353
FR5781	PRN-	ERS5513213	PRJEB42353
FR5845	PRN-	ERS5513214	PRJEB42353
FR5851	PRN-	ERS5513215	PRJEB42353
FR5875	PRN-	ERS5513216	PRJEB42353
FR5916	PRN-	ERS5513217	PRJEB42353
FR5938	PRN-	ERS5513218	PRJEB42353
FR5947	PRN-	ERS5513219	PRJEB42353
FR5952	PRN-	ERS5513220	PRJEB42353
FR5956	PRN-	ERS5513221	PRJEB42353
FR5982	PRN-	ERS5513222	PRJEB42353
FR5991	PRN-	ERS5513223	PRJEB42353
FR5998	PRN-	ERS5513224	PRJEB42353
FR5999	PRN-	ERS5513225	PRJEB42353
FR6024	PRN-	ERS5513226	PRJEB42353
FR6035	PRN-	ERS5513227	PRJEB42353
FR6037	PRN-	ERS5513228	PRJEB42353
FR6040	PRN-	ERS5513229	PRJEB42353
FR6041	PRN-	ERS5513230	PRJEB42353
FR6064	PRN-	ERS5513231	PRJEB42353
FR6069	PRN-	ERS5513232	PRJEB42353
FR6070	PRN-	ERS5513233	PRJEB42353
FR6080	PRN-	ERS5513234	PRJEB42353
FR6081	PRN-	ERS5513235	PRJEB42353
FR6083	PRN-	ERS5513236	PRJEB42353
FR6085	PRN-	ERS5513237	PRJEB42353
FR6093	PRN-	ERS5513238	PRJEB42353
FR6142	PRN-	ERS5513239	PRJEB42353
FR6143	PRN-	ERS5513240	PRJEB42353
FR6152	PRN-	ERS5513241	PRJEB42353
FR6160	PRN-	ERS5513242	PRJEB42353
FR6163	PRN-	ERS5513243	PRJEB42353
FR6164	PRN-	ERS5513244	PRJEB42353
FR6173	PRN-	ERS5513245	PRJEB42353
FR6174	PRN-	ERS5513246	PRJEB42353
FR6183	PRN-	ERS5513247	PRJEB42353

FR6194	PRN-	ERS5513248	PRJEB42353
FR6237	PRN-	ERS5513249	PRJEB42353
FR6238	PRN-	ERS5513250	PRJEB42353
FR6239	PRN-	ERS5513251	PRJEB42353
FR6241	PRN-	ERS5513252	PRJEB42353
FR6246	PRN-	ERS5513253	PRJEB42353
FR6247	PRN-	ERS5513254	PRJEB42353
FR6270	PRN-	ERS5513255	PRJEB42353
FR6305	PRN-	ERS5513256	PRJEB42353
FR6322	PRN-	ERS5513257	PRJEB42353
FR6323	PRN-	ERS5513258	PRJEB42353
FR6327	PRN-	ERS5513259	PRJEB42353
FR6350	PRN-	ERS5513260	PRJEB42353
FR6354	PRN-	ERS5513261	PRJEB42353
FR6356	PRN-	ERS5513262	PRJEB42353
FR6360	PRN-	ERS5513263	PRJEB42353
FR6361	PRN-	ERS5513264	PRJEB42353
FR6362	PRN-	ERS5513265	PRJEB42353
FR6365	PRN-	ERS5513266	PRJEB42353
FR6368	PRN-	ERS5513267	PRJEB42353
FR6374	PRN-	ERS5513268	PRJEB42353
FR6385	PRN-	ERS5513269	PRJEB42353
FR6386	PRN-	ERS5513270	PRJEB42353
FR6388	PRN-	ERS5513271	PRJEB42353
FR6390	PRN-	ERS5513272	PRJEB42353
FR6393	PRN-	ERS5513273	PRJEB42353
FR6394	PRN-	ERS5513274	PRJEB42353
FR6397	PRN-	ERS5513275	PRJEB42353
FR6398	PRN-	ERS5513276	PRJEB42353
FR6403	PRN-	ERS5513277	PRJEB42353
FR6415	PRN-	ERS5513278	PRJEB42353
FR6419	PRN-	ERS5513279	PRJEB42353
FR6424	PRN-	ERS5513280	PRJEB42353
FR6428	PRN-	ERS5513281	PRJEB42353
FR6462	PRN-	ERS5513282	PRJEB42353
FR6463	PRN-	ERS5513283	PRJEB42353
FR6466	PRN-	ERS5513284	PRJEB42353
FR6469	PRN-	ERS5513285	PRJEB42353
FR6470	PRN-	ERS5513286	PRJEB42353
FR6497	PRN-	ERS5513287	PRJEB42353
FR6510	PRN-	ERS5513288	PRJEB42353
FR6520	PRN-	ERS5513289	PRJEB42353
FR6522	PRN-	ERS5513290	PRJEB42353

FR6525	PRN-	ERS5513291	PRJEB42353
FR6527	PRN-	ERS5513292	PRJEB42353
FR6537	PRN-	ERS5513293	PRJEB42353
FR6538	PRN-	ERS5513294	PRJEB42353
FR6539	PRN-	ERS5513295	PRJEB42353
FR6548	PRN-	ERS5513296	PRJEB42353
FR6568	PRN-	ERS5513297	PRJEB42353
FR6573	PRN-	ERS5513298	PRJEB42353
FR6585	PRN-	ERS5513299	PRJEB42353
FR6589	PRN-	ERS5513300	PRJEB42353
FR6596	PRN-	ERS5513301	PRJEB42353
FR6600	PRN-	ERS5513302	PRJEB42353
FR6610	PRN-	ERS5513303	PRJEB42353
FR6616	PRN-	ERS5513304	PRJEB42353
FR6620	PRN-	ERS5513305	PRJEB42353
FR6625	PRN-	ERS5513306	PRJEB42353
FR6626	PRN-	ERS5513307	PRJEB42353
FR6632	PRN-	ERS5513308	PRJEB42353
FR6641	PRN-	ERS5513309	PRJEB42353
FR6643	PRN-	ERS5513310	PRJEB42353
FR6686	PRN-	ERS5513311	PRJEB42353
FR5869	PRN-	ERS1869864	PRJEB21744
FR5905	PRN-	ERS1869866	PRJEB21744
FR5910	PRN-	ERS1869867	PRJEB21744
FR5980	PRN-	ERS1869872	PRJEB21744
FR5990	PRN-	ERS1869875	PRJEB21744
FR6050	PRN-	ERS1869883	PRJEB21744
